# Longitudinal association between CSF markers of Alzheimer Disease and inflammation in preclinical Alzheimer Disease

**DOI:** 10.1002/alz.087640

**Published:** 2025-01-03

**Authors:** Isabel MacKenzie Sarty, Cynthia Picard, Anne Labonte, John C.S. Breitner, Judes Poirier

**Affiliations:** ^1^ Integrated Program in Neuroscience, Faculty of Medicine and Health Science, McGill University, Montreal, QC Canada; ^2^ Department of Psychiatry, McGill University, Montreal, QC Canada; ^3^ Douglas Mental Health Research Centre, Montreal, QC Canada; ^4^ Centre for Studies on Prevention of Alzheimer’s Disease (StoP‐AD Centre), Montréal, QC Canada; ^5^ Centre for Studies on Prevention of Alzheimer’s disease (StoP‐AD Centre), Douglas Mental Health Institute, Montreal, QC Canada; ^6^ Centre for Studies on Prevention of Alzheimer’s disease (StoP‐AD Centre), Montreal, QC Canada; ^7^ Department of Psychiatry, McGill University, Montréal, QC Canada; ^8^ Douglas Mental Health University Institute, Montréal, QC Canada; ^9^ Department of Medicine, McGill University, Montréal, QC Canada

## Abstract

**Background:**

Inflammation is central to Alzheimer Disease (AD), as astrocyte reactivity accompanies the appearance of Aβ and phosphorylated tau (Bellaver et al., 2023). As expected, therefore, AD patients have elevated levels of CSF inflammatory cytokines (Onyango et al., 2021). To understand the importance of these phenomena, exploration of individual inflammatory markers before and at the time of dementia onset is needed. To uncover key CK/Rs involved in the early pathogenesis of AD, we investigated selected cytokines and their receptors (CK/Rs) in the preclinical stages of the disease.

**Method:**

We analyzed 433 longitudinal CSF samples from 104 participants in the PREVENT‐AD cohort. These persons, who were symptom‐free at baseline but at enhanced risk of developing AD because of their family history, have now been followed for over 10 years. We used ELISA to analyze Aβ42, p181, and total tau in the CSF [Fujirebio Innotest, Ghent, Belgium], while CK/Rs were assayed using the Olink proximity extension assay Target 96 Inflammation panel [Upsala, Sweden]. Linear mixed model analyses controlled for participant age and APOE4 status.

**Result:**

We found that IL10RB, IL18, and CD40 levels in the CSF were significantly associated with both p(181)tau and Aβ42 levels in the CSF over time. IL10RB, IL18, and CD40 each showed significant positive association with p(181)tau levels across time (F(1,213.191) = 7.013, p = 0.009; F(1,237.624) = 9.972, p = 0.002; F(1,246.859) = 8.211, p = 0.005). The longitudinal effects of IL10RB and IL18 on Aβ42 were contingent on both sex and time (F(5,55.406) = 2.548, p = 0.038; F(5,54.792) = 4.280, p = 0.002), while the significantly positive association of CD40 levels on Aβ42 across time was stronger in females than in males (F(1, 59.669) = 4.099, p = 0.047).

**Conclusion:**

These investigations of CSF proteins in preclinical AD persons identified inflammatory markers that exhibited specific relationships with p(181)tau and Aβ42 across time and sex. These results may guide further study of biomarkers for detection of AD in its preclinical stages, and/or therapeutic strategies. Of special importance, characterization of the pathogenesis of pre‐symptomatic AD may facilitate development of early interventions for the prevention of AD dementia.

